# *Leishmania infantum* induces high phagocytic capacity and intracellular nitric oxide production by human proinflammatory monocyte

**DOI:** 10.1590/0074-02760190408

**Published:** 2020-04-17

**Authors:** Christiana Vargas Ribeiro, Bruna Fonte Boa Rocha, Edward Oliveira, Andrea Teixeira-Carvalho, Olindo Assis Martins-Filho, Silvane Maria Fonseca Murta, Vanessa Peruhype-Magalhães

**Affiliations:** 1 Fundação Oswaldo Cruz-Fiocruz, Instituto René Rachou, Grupo de Genômica Funcional e Proteômica de Leishmania spp. e Trypanosoma cruzi, Belo Horizonte, MG, Brasil; 2 Fundação Oswaldo Cruz-Fiocruz, Instituto René Rachou, Grupo Integrado de Pesquisas em Biomarcadores, Belo Horizonte, MG, Brasil

**Keywords:** L. braziliensis, L. infantum, monocyte subsets, lectins, cytokines, nitric oxide, surface glycoconjugates

## Abstract

**BACKGROUND:**

The mechanism of resistance to Sb^III^ in *Leishmania* is complex, multifactorial and involves not only biochemical mechanisms, but also other elements, such as the immune system of the host.

**OBJECTIVES:**

In this study, putative changes in the immunological profile of human monocytes infected with wild-type (WT) and antimony (Sb^III^)-resistant *Leishmania (Viannia) braziliensis* and *Leishmania (Leishmania) infantum* lines were evaluated.

**METHODS:**

Susceptibility assays WT and SbIII-resistant *L. braziliensis* and *L. infantum* were performed using lines THP-1 human monocytic lineage. Phagocytic capacity, cytokine profile, intracellular nitric oxide (NO) production and surface carbohydrate residues profile were performed in peripheral blood monocytes by flow cytometry.

**FINDINGS:**

The phagocytic capacity and intracellular NO production by classical (CD14^++^CD16^-^) and proinflammatory (CD14^++^CD16^+^) monocytes were higher in the presence of *L. infantum* lines compared to *L. braziliensis* lines. The results also highlight proinflammatory monocytes as the cellular subpopulation of major relevance in a phagocytosis event and NO expression. It is important to note that *L. infantum* induced a proinflammatory cytokine profile characterised by higher levels of TNF-α in culture supernatant than *L. braziliensis.* Conversely, both *Leishmania* lines induce high levels of IL-6 in culture supernatant. Analysis of the expression profile of surface carbohydrates showed that *L. braziliensis* presents 4.3-fold higher expression of galactose(β1,4)*N*-acetylglucosamine than *L. infantum* line. Interestingly, the expression level of α-*N*-acetylgalactosamine residues was 2-fold lower in the Sb^III^-resistant *L. braziliensis* line than its counterpart WT line, indicating differences in surface glycoconjugates between these lines.

**MAIN CONCLUSIONS:**

Our results showed that *L. braziliensis* and *L. infantum* induce different innate immune responses and a highly inflammatory profile, which is characteristic of infection by *L. infantum*, the species associated with visceral disease.

Leishmaniasis is caused by over 21 different species of unicellular protozoan parasites belonging to the genus *Leishmania*. It is classified as a neglected tropical disease, according to the World Health Organization (WHO), and is a public health issue in many developing countries.^1^ Leishmaniasis is characterised by a spectrum of clinical manifestations ranging from self-healing cutaneous (CL) and mucocutaneous (MCL) skin lesions to a visceral (VL) form, which is lethal if untreated. In the New World, *L. (L.) infantum* (syn. *L. (L.) chagasi*) is the causative agent of VL, whereas *L. (V.) braziliensis* causes CL and MCL.^2^

Many studies have been carried out to identify genes that may be involved in the N-methylglucamine antimoniate (Glucantime^®^)-resistance phenotype, first-choice drug for the treatment of human leishmaniasis in many countries.^3^ ^,^ ^4^ Among the mechanisms of drug resistance in *Leishmania* spp., the main one is the decrease in drug concentration in parasite.^5^ In addition, changes in the surface glycoconjugates expression profile of parasites have also been related to resistance and for this evaluation, lectins, which are glycoproteins of non-immunological origin, that interact in a reversible and specific way to carbohydrates or glycoconjugates are used. Studies involving an analysis of the surface glycoconjugates expression profile using specific lectins revealed that antimony-resistant *L. donovani* clinical isolates from patients in Bihar overexpress terminal glycoconjugates of *N*-acetyl-D-galactosamine residues.^6^ ^,^ ^7^

The mechanism of resistance to Sb^III^ in *Leishmania* is complex, multifactorial and involves not only biochemical mechanisms, but also other elements, such as the immune system of the host. A study using an isolate of *L. infantum*, with *in vitro* induced resistance to Sb^III^, reported resistance to NO and the cytotoxic effects of activated macrophages.^8^ Another study demonstrated that *L. braziliensis* promastigotes isolated from refractory patients are generally less susceptible to nitric oxide (NO).^9^ Moura et al.^10^ observed that Sb^III^-resistant isolates of *L. infantum* showed a higher rate of infectivity in macrophages and higher resistance to Sb^III^ and NO *in vitro* compared to wild-type (WT) samples. However, the involvement of the host immune system in the mechanisms of antimony-resistance in *Leishmania* requests to be better elucidated.

Clinical aspects and cure of leishmaniasis are related to the development of an effective and balanced immune response in the host. The intracellular parasite must be contained, whereas the immune response must be strictly controlled to avoid further tissue damage.^11^ ^,^ ^12^

The characteristics of the immunological responses developed during *Leishmania* sp. infections are quite variable among the affected individuals, resulting in several clinical aspects. These natural differences arise from factors such as the host’s immune status, the *Leishmania* species, parasite exposure, co-infections, among others.^11^ ^,^ ^12^

Immunity against *Leishmania* is mediated through a complex immunological parameters network, including the innate and adaptive immune response. The immune response plays an important role in the clinical cure of the disease or its progression.^11^ Thus, the presence of effector cells such as monocytes/macrophages, is critical for the control or development of leishmaniasis.^12^

Monocytes/macrophages are antigen presenting cells that represent one of the first steps in the innate immune response aimed at killing the *Leishmania* parasite. These cells use important mechanisms that modulate inflammatory response activation and kill the parasite by producing cytokines as TNF-α, and NO, reactive oxygen intermediates (ROI).^13^ ^,^ ^14^

Interestingly, *Leishmania* spp. modulates monocytes/macrophage functions by preventing oxidative burst and the effector functions that lead to its elimination and by improvement a bursting microenvironment of modulatory IL-4, IL-10, and TGF-β cytokines.^14^ ^,^ ^15^ ^,^ ^16^ ^,^ ^17^ ^,^ ^18^

A better understanding of the immune response against *Leishmania* spp. is very important to establishing a rational approach to chemotherapy and to comprehend mechanism of drug resistance. In this context, the objective of this study was to analyse changes in the immunological profile of monocytes after WT and Sb^III^-resistant *L. braziliensis* and *L. infantum* lines infection and evaluate the agglutination pattern these different parasite species by applying tests that assess the lectins’ binding specificity.

## SUBJECTS AND METHODS

*Human subjects* - The study population consisted of 10 non-infected volunteers. The individuals were five men and five women, ranging from 20 to 31 years of age. They did not report previous infection by *Leishmania* spp. All individuals live in Belo Horizonte, Minas Gerais, Brazil, and voluntarily agreed to donate blood samples for the research.

*Leishmania samples and culture conditions* - In this study, we used promastigote forms of *Leishmania (Viannia) braziliensis* (MHOM/BR/1975/2904) and *Leishmania (Leishmania) infantum* (MHOM/BR/1974/PP75) lines WT and resistant to potassium antimony tartrate (Sb^III^). The Sb^III^-resistant lines were previously selected *in vitro* by step-wise increase of Sb^III^ drug pressure.^19^ The parasites were grown at 26ºC in M199 medium supplemented with 2 mM L-glutamine, 5 μg/mL hemin, 50 μg/mL streptomycin, 2 μg/mL biopterin, 1 μg/mL biotin, 40 mM HEPES pH 7.4, 50 U/mL penicillin, and 10% v/v heat-inactivated fetal calf serum. All experiments were carried out with parasites in the stationary phase.

*Genotyping of Leishmania species using Hsp70 and ITS1 markers* - The WT and Sb^III^-resistant *L. braziliensis* and *L. infantum* lines were submitted to polymerase chain reaction-restriction fragment length polymorphism (PCR-RFLP) for genotyping analysis using specific primers for HSP70 and ITS1 genes, as previously described.^20^ Briefly, genomic DNA of these lines was obtained using the QIAquick DNA Extraction Kit (Qiagen), according to the manufacturer’s recommendations. After amplification, the PCR products were digested with the restriction endonuclease enzyme *Hae*III to analyse restriction fragments length polymorphisms (RFLP). Restriction profiles were analysed on 4% agarose GelRed (Biotium) stained and compared to the standard reference *Leishmania* species.

*Susceptibility assays WT and Sb* ^*III*^ *-resistant L. braziliensis and L. infantum lines* - THP-1 human monocytic lineage (ATCC TIB 202) were cultured in a complete RPMI-1640 medium [supplemented 10% foetal bovine serum (FBS), 2 mM glutamine, 100 U/mL penicillin, and 100 μg/mL streptomycin] and differentiated into macrophages by adding 20 ng/mL phorbol myristate acetate (PMA) in culture. Macrophages (4 x 10^5^ cells/well) were seeded in 24 well cell culture plates containing 13 mm round coverslips and incubated for 72 h at 37ºC in a humid atmosphere containing 5% CO_2_. Then, the adhered macrophages were exposed to promastigote forms of WT and Sb^III^-resistant *L. braziliensis* and *L. infantum* lines at the stationary phase (4 x 10^6^ parasites/well) (10:1 parasites/macrophage). After 5 h of infection, the free parasites were removed and a RPMI-1640 medium was added in the absence or presence of Sb^III^ at a concentration ranging from 12.5 to 200 µM. After 72 h of incubation, the adhered macrophages were stained by panoptic staining. The infected cells and the number of intracellular amastigotes were determined using ImageJ software (1.50i version, Wayne Rasband National Institute of Health). EC_50_ values were obtained from three independent measurements in triplicate, using the linear interpolation method.

*L. braziliensis and L. infantum Alexa Fluor 647-labelling procedures* - Promastigote forms of WT and Sb^III^-resistant *L. braziliensis and L. infantum* lines were obtained from cultures in a M199 medium supplemented with 10% FBS, maintained at 26ºC in BOD incubator for five and seven days, respectively. The parasites were stained with Alexa Fluor 647 at a final concentration of 3.2 µg/mL at 37ºC for 45 min in a 5% CO_2_ incubator. After the Alexa Fluor 647-labelling procedure, parasites were washed once with phosphate buffered saline (PBS) pH 7.4 and resuspended in PBS supplemented with 10% FBS. The Alexa Fluor 647-labelled parasite suspension was adjusted to 1 x 10^8^/mL and maintained at 26ºC in a BOD incubator until use. Aliquots of Alexa Fluor 647-labelled parasites were run in LSR Fortessa (BD) to evaluate the efficiency of the Alexa Fluor 647-labelling procedure. The ideal Alexa Fluor 647-labelled parasite staining would lead to a single peak around 10^3^ and 10^4^ log intervals in histogram plots.

*In vitro L. braziliensis and L. infantum promastigote peripheral blood monocyte infection* - Human heparinised whole blood was centrifuged at 800 x g for 10 min at 18ºC. The plasma was removed, the whole blood cells (erythrocytes and leukocytes) washed two times with RPMI 1640 medium, and the final cell suspension adjusted to 1 x 10^7^ cells/mL. For the Alexa Fluor 647-labelled promastigotes platform, 1 x 10^7^ leukocytes were incubated with 1 mL of RPMI 1640 medium and 50 µL of Alexa Fluor 647-labelled WT or Sb^III^-resistant *L. infantum* or *L. braziliensis* promastigotes at 5 x 10^6^ parasites/mL. Short-term *in vitro* cultures were performed in five distinct platforms for promastigote forms referred to as “uninfected culture”, “infected WT promastigote of *L. infantum* culture”, “infected Sb^III^-resistant promastigote of *L. infantum* culture”, “infected WT promastigote of *L. braziliensis* culture”, and “infected Sb^III^-resistant promastigote of *L. braziliensis* culture” by 5 h of incubation under gentle shaking in an orbital shaker, at 37ºC in a 5% CO_2_ incubator. After incubation, each tube was centrifuged at 600 x g for 7 min at 18ºC. The supernatants were maintained for 1 month at -70ºC for cytokine analysis.

*Intracellular NO expression in peripheral blood monocytes after WT and Sb* ^*III*^ *-resistant L. braziliensis and L. infantum in vitro infection* - The NO expression was performed using peripheral blood monocytes as previously reported.^14^ ^,^ ^21^ Membrane-permeable fluorescent NO indicator, DAF-2DA is deacetylated by intracellular esterases to DAF-2 that reacts with NO to yield the highly fluorescent, a membrane-impermeable compost, the triazolofluorescein (DAF-2T), allow to distinguish between NO generated inside the monocytes and NO-derived from an exogenous source. The culture was performed using 1 x 10^7^ leukocytes incubated with 5 µL of WT or Sb^III^-resistant *L. braziliensis* or *L. infantum* Alexa Fluor 647-labelled promastigotes for 1 h at 37ºC in a 5% CO_2_ incubator. A control culture (“uninfected culture”) was used to determine the basal levels of intracellular NO. The samples were then incubated in the presence of 4,5-diaminofluorescein diacetate (DAF-2DA) (10 µM, Sigma, MO, USA) for 3 h at 37ºC in a 5% CO_2_ incubator. The samples were labelled for 20 min at room temperature in the presence of PerCP-labelled anti-CD16 mAb, V450-labelled anti-CD14 mAb and PE-labelled anti-HLA-DR mAb. Following incubation, the red blood cells were lysed with FACS lysing solution (Becton Dickinson, CA, USA) and the cell pellet was washed and centrifuged at 600 x g for 7 min at room temperature and resuspended with PBS solution for immediate acquisition in the LSR Fortessa cytometer. A total of 3,000 events/tube on CD14^+^ monocyte gate was acquired. In order, to evaluate the enzymatic origin of the monocytes NO detected, the selective iNOS inducer [lipopolysaccharide (LPS) 10 µg/mL, Sigma, USA] and inhibitor (Aminoguanidine-AG 10mM, Sigma, MO, USA) were used. The LPS and AG cultures were used as functional specificity controls associated with monocytes NO production by iNOS pathway in all experiments. The data were analysed using Flow Jo 10.1 software.

*Detection of supernatant cytokine levels by cytometric bead array* - The cytometric bead array (CBA) is a method in which the simultaneous measurement of multiple cytokines is performed in a single sample. In this study, culture supernatant aliquots were used to quantify the secreted cytokine levels using CBA Human Inflammatory kit (Becton Dickinson, San Diego, CA, USA) for evaluation of IL-8, IL-1b, IL-6, IL-10, TNF-a, IL-12 p70 (limit of detection: 3.6 pg/mL, 7.2 pg/mL, 2.5 pg/mL, 3.3 pg/mL, 3.7 pg/mL and 1.9 pg/mL, respectively). Brieﬂy, 25 µL of supernatants or standards were added to 17 µL of mixture beads and incubated for 3 h at room temperature in the dark. After incubation, the samples and standards were washed with 500 µL of wash buffer and centrifuged at 600 x g for 7 min at room temperature. Subsequently, 17 µL of detection cocktail consisting of mix PE-conjugated mAbs were added to each tube and the mixture re-incubated for 180 min at room temperature in the dark. Following incubation, the samples and standards were washed again with 500 µL of wash buffer and centrifuged at 600 x g for 7 min at room temperature. After washing, 250 µL of wash buffer were added to each tube prior to data acquisition (300 events/tube) using FACSVerse ﬂow cytometer (Becton Dickinson, San Diego, CA, USA). Data analysis was performed using FCAP Array 3.1 software (Becton Dickinson, San Diego, CA, USA). Secreted cytokines concentrations were expressed as pg/mL and the results plotted in bar graphs presenting mean + standard error of the mean (SEM) in log scale.

*Expression profile of surface carbohydrates using Erythrina cristagalli, Dolichos biflorus, and Concanavalin A lectins in WT and Sb* ^*III*^ *-resistant L. braziliensis and L. infantum lines* - The *Erythrina cristagalli* (ECA), *Dolichos biflorus* (DBA), and Concanavalin-A (Con-A) lectins were used to compare the expression profile of the surface carbohydrates in WT and antimony-resistant *L. braziliensis* and *L. infantum* lines, as they have different carbohydrate binding specificities. ECA binds to residues of galactose (β1,4) *N*-acetylglucosamine, DBA recognises *α-N*-acetylgalactosamine residues, and Con-A binds to terminal portions of *α*-D-mannose and α-D-glucose residues. Promastigote forms were incubated separately with each of these lectins conjugated to fluorescein isothiocyanate (FITC). Briefly, promastigote forms of the WT and Sb^III^-resistant *L. braziliensis* and *L. infantum* lines in the stationary growth phase (2 x 10^6^ parasites/mL) were washed with PBS and incubated with each lectin conjugated to FITC: ECA and DBA (EY Laboratories, INC, San Mateo, CA, USA) and Con A (Vector Laboratories, Burlingame, CA, USA) at a final concentration of 10 mg/mL for 30 min at 37ºC in a 5% CO_2_ incubator. The total of 30.000 parasites/tube were then acquired to flow cytometer (LSR Fortessa) and the data were analysed using Flow Jo v.10 software. The geometric mean fluorescence intensity (gMFI) and the Con-A labelled-*Leishmania* percentage (%) for each *L. braziliensis* and *L. infantum* sample was determined.

*Statistical analysis* - Statistical analyses were performed using GraphPad Prism software (San Diego, USA, version 5.00). First, the Kolmogorov-Smirnov test was performed as normality test to evaluate the Gaussian distribution of data. The unpaired *t* test was used for comparative analyses between two groups, whereas, the one-way analysis of variance followed by Tukey’s *post-test* were used for comparative analyses among three or more groups. For susceptibility assays, bidirectional analysis of variance (ANOVA two-way) followed by the Bonferroni post-test was used. In all cases, the differences were considered statistically significant when the *p* value was less than 0.05.

*Ethics* - This work complied with resolution number 466/2012 from the National Health Council for research involving human subjects and was approved by the Ethical Committee at Instituto René Rachou (CEPSH/IRR/FIOCRUZ protocol:1.368.058), Belo Horizonte, Minas Gerais, Brazil.

## RESULTS

*Genotyping of Leishmania lines using Hsp70 and ITS1 markers* - The ITS1 and Hsp70 molecular markers for genotyping were used to confirm the *Leishmania* species used in this study, of WT and Sb^III^-resistant *L. braziliensis* and *L. infantum* lines. Both pairs of Hsp70 and ITS1 primers amplified fragments of 1300 and 300-350 bp, respectively, in all *Leishmania* species analysed. The restriction profiles of the PCR products were compared to the standard reference *Leishmania* species: *L. amazonensis* (IFLA/BR/67/PH8), *L. braziliensis* (MHOM/BR/75/M2903), *L. infantum* (MHOM/BR/74/PP75), and *L. guyanensis* (MHOM/BR/75/M4147). The results confirmed that the *Leishmania* species used in this work correspond to *L. braziliensis* and *L. infantum* [Supplementary data (Figure)].

**Fig. 1:**
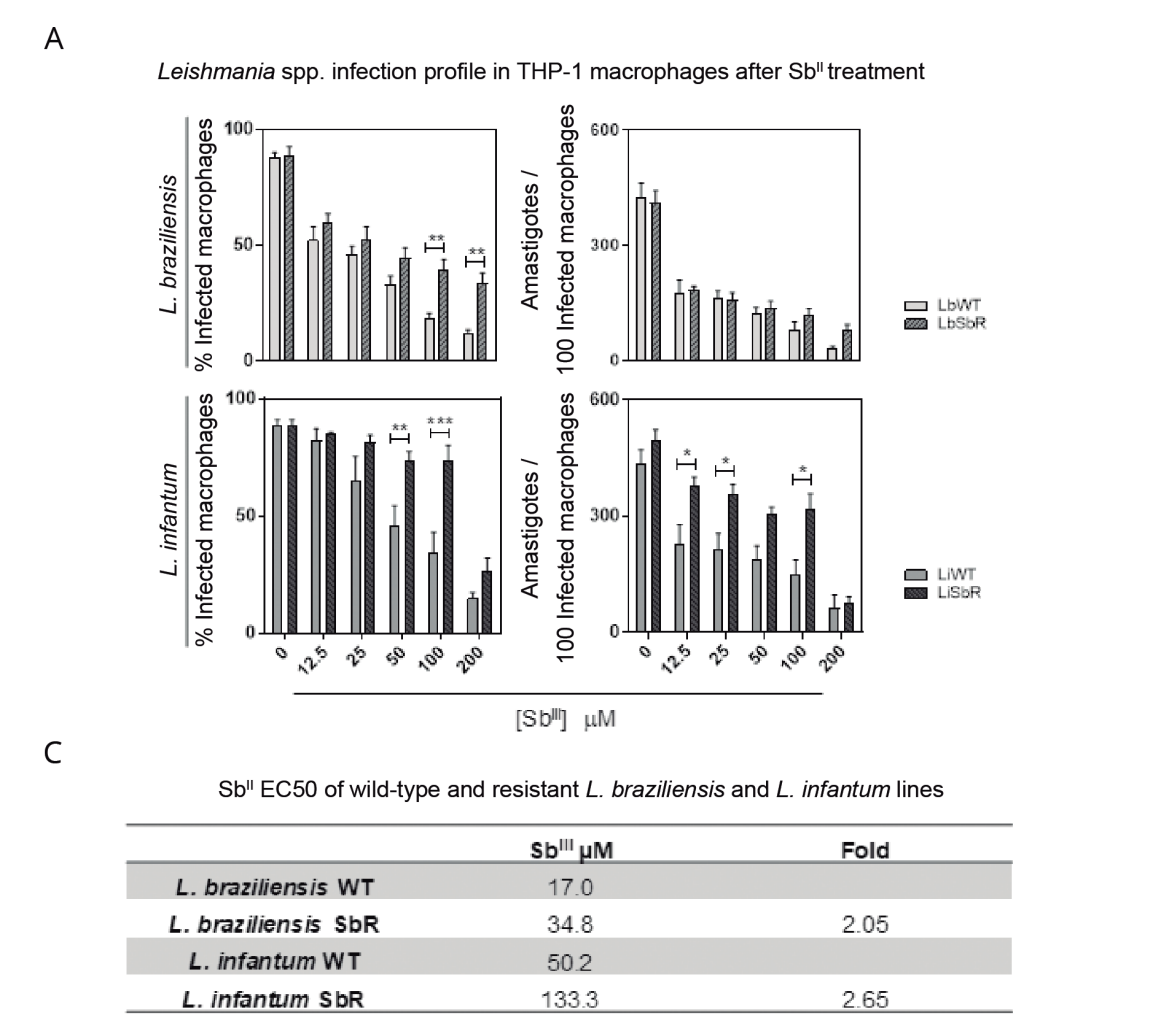
Sb^III^ susceptibility was evaluated on amastigote forms using THP-1-derived-human macrophages. (A) THP-1 macrophages infected with wild-type (WT) (LbWT) and Sb^III^-resistant (LbSbR) *Leishmania braziliensis* lines (top graph) or WT (LiWT) and Sb^III^-resistant (LiSbR) *L. infantum* lines (bottom graph) were cultured in the absence or presence of increasing Sb^III^ concentrations (12.5 to 200 µM) for 72 h and the percentage of infected macrophages and number of parasites per 100 macrophages were determined. Sb^III^-resistant lines were compared with their respective WT counterparts and the statistically significant differences (*p < 0.05, **p < 0.01, ***p < 0.001) were represented by connector lines. (B) Resistance index of the EC_50_ value for LbSbR and LiSbR compared to the EC_50_ value for LbWT and LiWT.

*Susceptibility assays of WT and Sb* ^*III*^ *-resistant L. braziliensis and L. infantum lines using THP-1 macrophages* - To analyse whether the Sb^III^-resistant phenotype persists in parasite intracellular forms, amastigote forms of WT and Sb^III^-resistant *L. braziliensis* and *L. infantum* lines were submitted to susceptibility assays with Sb^III^. The data showed that the numbers of infected macrophages were higher in the Sb^III^-resistant lines of both *Leishmania* species than in their WT counterparts (Fig. 1A). The number of amastigotes/100 infected macrophages was also higher in the Sb^III^-resistant than in the WT *L. infantum* line. The amastigote forms from both Sb^III^-resistant *L. braziliensis* and *L. infantum* lines were about 2.05 and 2.65-fold more resistant to Sb^III^ (EC_50_ 34.8 and 133.3 µM for LbSbR and LiSbR, respectively) than to their respective counterpart WT lines (EC_50_ 17 and 50.2 µM for LbWT and LiWT) (Fig. 1B).

**Fig. 2:**
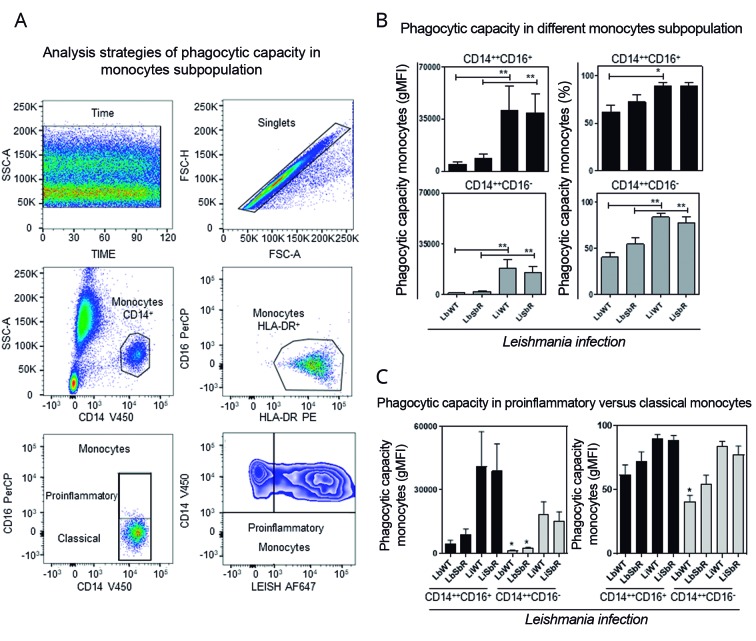
(A) flow cytometric analysis of monocyte subpopulations. First, the quality of the acquisition was estimated in Time *versus* Side Scatter-A graphs (SSC-A, granularity). Then, the selection of singlets was performed in Forward Scatter (FSC-A, size) *versus* FSC-H. Discrimination of the monocyte population was possible in anti-CD14-V450 *versus* SSC-A plot. Classical (CD14^++^CD16^-^) and proinflammatory (CD14^++^CD16^+^) monocyte subpopulations were plotted in anti-CD14-V450 *versus* anti-CD16-PerCP graphs, and the evaluation of the phagocytic capacity was obtained in anti-*Leishmania* AF647^+^ *versus* anti-CD14-V450 graphs to classical (CD14^++^CD16^-^) and proinflammatory (CD14^++^CD16^+^) monocyte subpopulations. An illustrative graph of phagocytic capacity of proinflammatory (CD14^++^CD16^+^) monocyte was presented in the last image. (B) Analysis of the phagocytic capacity of CD14^++^CD16^-^ and CD14^++^CD16^+^ monocytes of healthy volunteers were shown in bar graphs presenting mean + SEM, after incubation with wild-type (WT) and Sb^III^-resistant *L. braziliensis* and *L. infantum* lines. The results were presented by percentage (%) and geometric mean fluorescence intensity (gMFI); statistically significant differences (*P < 0.05, **P < 0.01) between WT and Sb^III^-resistant *L. braziliensis* and *L. infantum* lines were shown by connector lines. (C) Analysis of the phagocytic capacity of CD14^++^CD16^-^ *versus* CD14^++^CD16^+^ monocytes of healthy volunteers were shown in bar graphs presenting mean + SEM, after incubation with WT and Sb^III^-resistant *L. braziliensis* and *L. infantum* lines. Statistically significant differences (*P* < 0.05) between the promastigote of WT and Sb^III^-resistant *Leishmania* lines in different monocyte subpopulations were shown by asterisks (*).

*Phagocytic capacity of proinflammatory and classical monocytes* - In order to evaluate the phagocytic capacity (percentage of monocytes Leish^+^) of peripheral blood proinflammatory/ (CD14^++^CD16^+^) and classical (CD14^++^CD16^-^) monocytes, they were infected with WT and Sb^III^-resistant *L. braziliensis* and *L. infantum* lines and evaluated by flow cytometry using phenotypic-specific selection strategy (anti-HLA-DR PE, anti-CD14 V450, and anti-CD16 PerCP antibodies) (Fig. 2A). The results showed that the phagocytic capacity of proinflammatory and classical monocytes was higher for WT and Sb^III^-resistant *L. infantum* lines as compared to *L. braziliensis* lines (Fig. 2B). Moreover, our data highlight proinflammatory monocytes as the cellular subpopulation of major relevance in a phagocytosis event (Fig. 2C). A second approach was used as complementary analysis with the purpose to evaluate the infection density of the monocytes (gMFI of infected cells). Our data demonstrated that despite the high percentage of *L. braziliensis* ^+^ proinflammatory monocytes (above 50% of infected cells), the number of parasites internalised or present in the cell membrane (interaction) is small as compared to *L. infantum* challenge (Fig. 2B - top graphs). On the other hand, no differences were found between Sb^III^-resistant parasites as compared to WT of the same *Leishmania* spp.

Functional biomarkers evaluation after WT and Sb^III^-resistant promastigote *L. braziliensis* and *L. infantum* infection

**Fig. 3:**
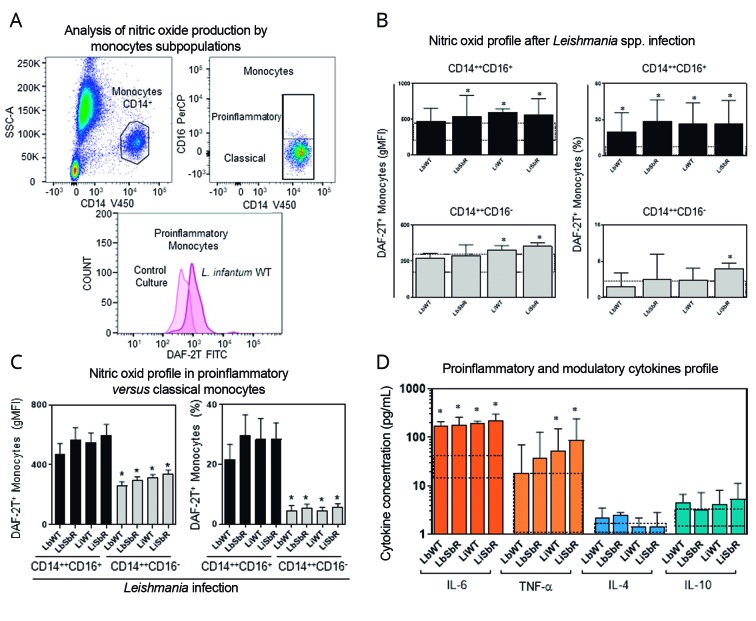
(A) nitric oxide (NO) strategy analysis in monocytes. Discrimination of the monocyte population was possible in anti-CD14-V450 *versus* SSC-A plot. Classical (CD14^++^CD16^-^) and proinflammatory (CD14^++^CD16^+^) monocyte subpopulations were plotted in anti-CD14-V450 *versus* anti-CD16-PerCP graphs, and the intracellular NO expression was obtained in a DAF-2T FITC *versus* count histogram. (B) Analysis of the intracellular NO expression by CD14^++^CD16^-^ and CD14^++^CD16^+^ monocytes of healthy volunteers were shown in bar graphs presenting mean + SEM, after incubation with promastigotes of wild-type (WT) and Sb^III^-resistant *Leishmania braziliensis* and *L. infantum* lines. The control culture (basal NO expression) was shown by dashed rectangle (----) presenting the 95% confidence interval (95%CI). Statistically significant differences (p < 0.05) between promastigote of WT and Sb^III^-resistant *Leishmania* lines as compared to control culture were shown by asterisks (*). (C) Analysis of the intracellular NO expression by CD14^++^CD16^-^ *versus* CD14^++^CD16^+^ monocytes of healthy volunteers were shown in bar graphs presenting mean + SEM, after incubation with promastigotes of WT and SbIII-resistant *L. braziliensis* and *L. infantum* lines. The results were presented by percentage (%) and geometric mean fluorescence intensity (gMFI). Statistically significant differences (p < 0.05) between promastigotes of WT and Sb^III^-resistant *Leishmania* lines in different monocyte subpopulations were identified by asterisks (*). (D) Secreted cytokine profile was performed in culture supernatant of whole blood after promastigotes of WT and Sb^III^-resistant *L. braziliensis* and *L. infantum* lines infection using flow cytometric CBA immunoassay. Secreted IL-6, TNF-a, IL-4, and IL-10 concentrations were expressed as pg/mL and the results plotted in bar graphs presenting mean + SEM in log scale. The control culture (basal secreted cytokines) was shown by dashed rectangle (----) presenting the 95%CI. Statistically significant differences (p < 0.05) between promastigotes of WT and Sb^III^-resistant *Leishmania* lines cultures and control culture were shown by asterisks (*).

*Intracellular NO expression by proinflammatory and classical monocytes* - The reagent 4,5-diaminofluorescein diacetate (DAF-2DA) was used to evaluate intracellular NO expression. The monocytes were evaluated by flow cytometry in anti-CD14 V450 *versus* anti-CD16 PerCP plots, and NO expression was evaluated in histogram graphs highlighting the displacement of DAF-2T FITC in X axis (Fig. 3A). NO inducer (LPS) and NO inhibitor (AG) stimuli were used as functional and specificity controls, respectively. The control culture and WT and Sb^III^-resistant *L. braziliensis* and *L. infantum* promastigote cultures were used to evaluate the basal and *Leishmania*-specific NO production by proinflammatory and classical monocytes. The LPS data presented mean + SEM equal to 62.29 ± 8.45 (% - proinflammatory monocytes) and 24.23 ± 8.21 (% - classical monocytes). Already AG analyses showed mean + SEM equal to 6.69 ± 3.37 (% -proinflammatory monocytes) and 3.22 ± 0.35 (% - classical monocytes), indicative of perfect protocol controls. Data analyses were performed in comparison with control culture (basal cytokine profile - dashed rectangle presenting the 95%CI; Fig. 3B). The results showed that Sb^III^-resistant *L. braziliensis* and both WT and Sb^III^-resistant *L. infantum* lines induced a statistically significant increase in fluorescence intensity and percentage of DAF-2T^+^ proinflammatory monocytes (p < 0.05; Fig. 3B - Top Graphs). Interestingly, our data demonstrated a significant increase in fluorescence intensity and percentage of classical DAF-2T^+^ monocytes subpopulation after *L. infantum* stimulation (Fig. 3B - Bottom Graphs). It also highlighted proinflammatory monocytes as the principal source of NO as compared to classical monocytes (Fig. 3C). No differences were found between Sb^III^-resistant parasites as compared to WT of the same *Leishmania* spp.

*Proinflammatory and modulatory cytokine profile in culture supernatant* - The Cytometric Bead Array (CBA) system was used to quantify cytokine supernatant levels. Promastigote forms of all *Leishmania* lines analysed induced high levels of proinflammatory IL-6 cytokine as observed in the supernatants of the different culture conditions. Data analyses were performed in comparison with control culture [basal cytokine profile - dashed rectangle presenting the 95% confidence interval (95%CI); Fig.3D]. Interestingly, both WT and Sb^III^-resistant *L. infantum* lines also induced higher levels of TNF-α (p < 0.05; Fig. 3D). The data did not show differentiated expression of modulatory cytokines, IL-4 and IL-10, in the culture conditions (Fig. 3D).

**Fig. 4:**
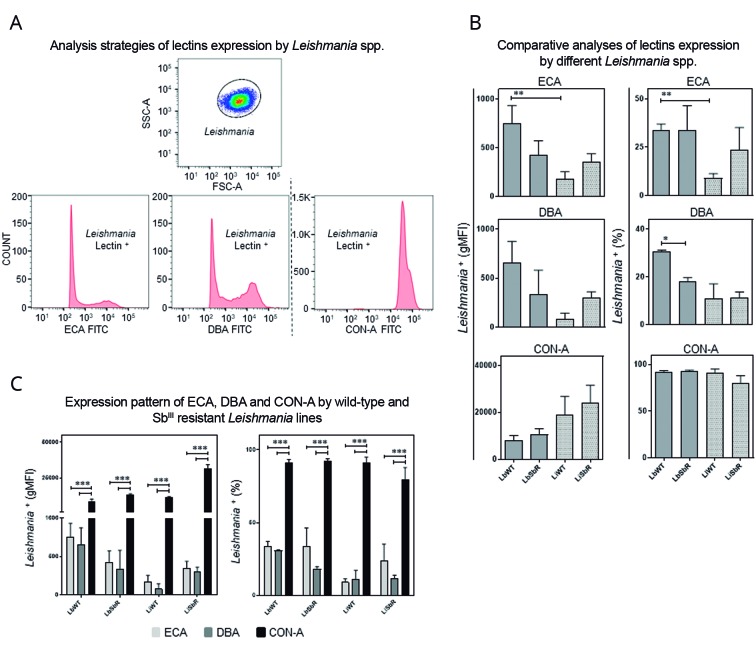
flow cytometric analysis of the differential expression of surface carbohydrates in wild-type (WT) and Sb^III^-resistant *Leishmania* lines using *Erythrina cristagalli* (ECA), *Dolichos biflorus* (DBA), and Concanavalin-A (Con-A) lectins conjugated to fluorescein isothiocyanate (FITC). ECA binds to galactose (β 1,4) *N*-acetylglucosamine residues, DCA recognises α-*N*-acetylgalactosamine residues, and Con-A binds to terminal portions of α-D-mannose and α-D-glucose residues. (A) The *Leishmania* spp. population was selected in SSC-A *versus* FSC-A graph, and ECA, DBA and Con-A lectin profiles were performed in lectin-FITC *versus* count histograms. (B) Comparison of the ECA, DBA, and Con-A lectins binding profiles were presented in bar graphs showing the mean + SEM for WT and Sb^III^-resistant *L. braziliensis* and *L. infantum* lines. The results were presented by percentage (%) and geometric mean fluorescence intensity (gMFI), and statistically significant differences (*p < 0.05; **p < 0.01) between Sb^III^-resistant *L. braziliensis* and *L. infantum* lines were shown by connector lines. (C) Evaluation of the ECA, DBA, and Con-A lectins binding profiles for each *Leishmania* spp. line was presented in bar graphs showing the mean + SEM. Statistically significant differences (***p < 0.001) between Con-A expression and the other lectins in WT and Sb^III^-resistant *L. braziliensis* and *L. infantum* lines were shown by connector lines.

*Evaluation of surface carbohydrate residues in L. braziliensis and L. infantum lines* - We also investigated the expression profile of the surface carbohydrates galactose (*β* 1,4) *N*-acetylglucosamine (ECA), *N-*acetylgalactosamine (DBA), and Concanavalin-A (Con-A) in the WT and Sb^III^-resistant lines of each species. The *Leishmania* parasites were selected in SSC-A *versus* FSC-A graphs and the surface carbohydrates profile was performed by lectin-FITC expression (Fig. 4A). The results showed that the WT *L. braziliensis* line (LbWT) expresses 4.3-fold more galactose (*β* 1,4) *N*-acetylglucosamine residue compared to the WT *L. infantum* line (LiWT) (ECA-Lectin^+^). In addition, a 2-fold lower expression of *N-*acetylgalactosamine residue (DBA-Lectin^+^) was detected in the Sb^III^-resistant *L. braziliensis* as compared to its WT counterpart (Fig. 4B). A similar parasite percentage Con-A-labelled for each *Leishmania* sample was observed, indicating the homogeneity of the parasites regarding the expression of molecules containing mannose or glucose residues (Fig. 4C). It is important to emphasize that regardless of antimony-resistance phenotype, α-D-mannose and α-D-glucose are the most prevalent carbohydrates in the membrane of parasites compared to other evaluated.

## DISCUSSION

Parasites of the genus *Leishmania* appear to be related to escape mechanisms that repress the normal functions of the macrophage, an immune system effector cell.^11^ ^,^ ^12^ Monocytes/macrophages are antigen-presenting cells that act as parasite hosts and are responsible for eliminating them. Phagocytosed parasites are an important mechanism modulating the activation of the inflammatory response and controlling their growth.^11^ ^,^ ^12^ According to the phenotypic-functional properties, the monocytes population is segregated into classical (CD14^++^CD16^-^), proinflammatory (CD14^++^CD16^+^) and patrolling (CD14^dim^CD16^+^) monocytes.^22^ The classical monocyte subset shows a high expression of genes associated with tissue repair, anti-apoptotic properties, and acts as the first line of defense of the innate immune response against intracellular pathogens due to its phagocytic capacity. Proinflammatory monocytes express cytoskeletal rearrangement genes at high levels, presenting high phagocytosis capacity, anti-proliferative and pro-apoptotic properties, and produce higher levels of proinflammatory cytokines.^23^

Previous studies have shown that a good Type 1 immune response (proinflammatory profile) is required, aiding in parasite protection. If the response is Type 1, cytokines such as IL-2, IFN-g, TNF-a, and IL-12 will be produced, promoting the activation of macrophages, NO synthesis, and parasite destruction. Conversely, if the response is Type 2, IL-4 and IL-10 will be produced, which cause a low macrophage activation and, consequently, clinical forms will appear.^11^ ^,^ ^12^ ^,^ ^24^ Several *Leishmania* species have the ability to suppress the response to infection through inhibition of NO synthesis and proinflammatory cytokine production.^11^ ^,^ ^12^ ^,^ ^24^ However, recent literature has showed that *L. braziliensis-*induced infection has been characterised by an inflammatory response. Cytokines such as IL-6, IL-1β, IL-17 and TNF-a have been associated with exacerbation of the infection, with TNF-a, an important NO inducer, being particularly significant in mucosal leishmaniasis evolution.^24^ In VL, the increased production of inflammatory cytokines without parallel infection control has also contributed to pathogenesis. Several human VL studies have demonstrated elevated serum levels of IL-4, IL-6, IL-10, IL-12, IL-13, IFN-g, and TNF-a in active disease compared to asymptomatic infection.^15^ ^,^ ^16^ ^,^ ^25^ In this sense, the progression/worsening of the clinical status would be promoting a lack of control of the inflammatory response and this “cytokine storm” without modulation favors the systemic inflammatory syndrome in VL patients and disfiguring mucosal lesions.^25^ ^,^ ^26^ On the other hand, as in other infectious and parasitic diseases, individuals who development efficient immunological response to the infection and do not presented the apparent disease allows to understand the immunological mechanisms of protection against infection. These individuals present satisfactory balance between modulatory and inflammatory biomarkers. In Tegumentary Leishmaniasis the subclinical infection is characterised by a lower IFN-g and TNF-a production, suggesting that this modulated immune response has the ability to control infection without causing pathology.^27^ Similarly, asymptomatic VL individuals presented basal profile cytokine.^15^ ^,^ ^16^ ^,^ ^25^

In this study, the main objective was to analyse changes in the immunological profile of human peripheral blood monocytes after infection with WT and Sb^III^-resistant *L. braziliensis* and *L. infantum* lines. Our data demonstrated that *L. infantum* induced higher levels of TNF-a compared to *L. braziliensis.* However, both *Leishmania* species induce high levels of IL-6 in monocyte culture supernatants. Our results showed that *L. infantum* induced a higher phagocytic capacity, associated with NO and TNF-a production by human monocytes as compared to *L. braziliensis*. However, our group, in cutaneous leishmaniasis, showed protective immune response associate with CD23-IgE-mediated NO release, iNOS specific and increase of intracellular NO after treatment.^14^ ^,^ ^18^ Thus, our data are in concordance with literature studies.

Regarding cytokine profile, although we not determine the source, we believed that the monocytes were the main cell population associated with this cytokine profile because they are physiologically the peripheral blood cells that most contribute with TNF-a and IL-6 production. Our data of proinflammatory monocytes reinforce this hypothesis since proinflammatory monocytes are highlighted as the cellular subpopulation of greater relevance in TNF-a and IL-6 production. In our study, we observed increase of *Leishmania* phagocytosis, specially *L. infantum* and NO expression and suggest that the monocytes are the main responsible by the TNF-a and IL-6 production.

This interspecific difference showing that *L. infantum* induced greater phagocytic capacity and production of NO and TNF-a by human monocytes compared to *L. braziliensis.* In the literature, studies point to the existence of polymorphisms in the structure of LPG intra and interspecies of *Leishmania*.^28^ ^,^ ^29^ Polymorphisms of the glycoconjugates (LPG, GIPLs and gp63), are important in the differential regulation of the initial events of the immune response, as well as in the establishment of the infection. The variation in LPG structure results in a differential activation of macrophages, resulting in distinct production of cytokines and/or NO levels.^29^ The presence of genetic diversity among lines allows a distinct ability to infect macrophages and thus the parasite surface molecules can modulate the interaction between *Leishmania* and host cells, influencing the clinical course of the disease^30^ and on the appearance of different immunopathology in leishmaniasis.^28^ As mentioned, discrepancies in the structure and composition of these molecules can influence the innate immune response and the differential establishment of the infection, generating a variety of clinical manifestations, such as LV and LT.^28^

Interesting, no difference was observed in the phagocytic capacity and NO/cytokine production by human monocytes between WT and Sb^III^-resistant *L. braziliensis* and *L. infantum* lines. The resistant lines studied here have a low antimony-resistance index (2.0 and 2.65-fold) in THP-1 macrophages. We believe that if this resistance index had been higher, it would possibly alter the phagocytic capacity and other complementary parameters evaluated in the study. The Sb^III^-resistance mechanism in *Leishmania* is complex, multifactorial, and involves not only biochemical mechanisms but also other elements, such as the host immune system. Literature data using other antimony-resistant models have revealed some differences in *Leishmania* lines. Holzmuller et al.^8^ reported that an isolate of *L. infantum* with resistance induced *in vitro* to Sb^III^ was resistant to NO donors and the cytotoxic effects of activated macrophages. It has also been shown that *L. braziliensis* promastigotes isolated from refractory patients are generally less susceptible to NO.^9^ Moura et al.^10^ observed that antimony-resistant *L. infantum* isolates had a higher rate of infectivity in macrophages and a greater resistance to Sb^III^ and NO *in vitro* as compared to WT samples. The authors further suggested that clinical isolates of *L. infantum* resistant to Sb^III^ may induce the production of inflammatory cytokines and inhibit the mechanisms associated with macrophage death.

We also investigated the expression profile of the surface carbohydrates galactose (*β* 1,4) *N*-acetylglucosamine, using *Erythrina cristagalli* lectin (ECA), and *N*-acetylgalactosamine, using *Dolichos biflorus* (DBA) in the WT and Sb^III^-resistant *L. braziliensis* and *L. infantum* lines. The data showed a higher expression of galactose (β1,4) *N*-acetylglucosamine in WT *L. braziliensis* than in *L. infantum* line, indicating interspecific differences of the surface carbohydrates. The DBA expression profile showed that the Sb^III^-resistant *L. braziliensis* line expresses lower α*-N*-acetylgalactosamine residues than its counterpart WT line. In contrast, Mukhopadhyay et al.,^7^ using the same ECA and DBA lectins, showed that antimony-resistant *L. donovani* isolates overexpress surface glycoconjugates containing *N*-acetylgalactosaminyl residues. This difference may be due to different *Leishmania* species analysed. Drug resistance mechanism is complex, multifactorial and varies among the different *Leishmania* species analysed. The role of these surface glycoconjugates in the antimony-resistance phenotype should be further investigated. Concanavalin-A was used as a control and our data also showed the homogeneity of parasite populations in both species regarding the expression of molecules containing mannose and glucose residues, possibly LPG, since approximately 90% of the parasites were labelled with Con-A.

In this study, we used promastigote forms of only one strain of each *Leishmania* species: *L. braziliensis* (2904) and *L. infantum* (PP75), then further studies analysing a greater number of strains are need to support better the results.

In conclusion, our results showed that the phagocytic capacity and intracellular NO and TNF-a cytokine production by human monocytes were higher in the presence of *L. infantum* lines as compared to *L. braziliensis* lines. In addition, the data also highlighted proinflammatory monocytes as the cellular subpopulation of major relevance in phagocytosis events and NO expressions.
