# Supplementary material for: Leishmania infantum induces high phagocytic capacity and intracellular nitric oxide production by human proinflammatory monocyte
Source: Mem Inst Oswaldo Cruz. 2020 Apr 17;115:e190408. doi: 10.1590/0074-02760190408 (PMC7164402; doi:10.1590/0074-02760190408)
Supplement: Supplementary file 1 [file 1678-8060-mioc-115-e190408-s.pdf]

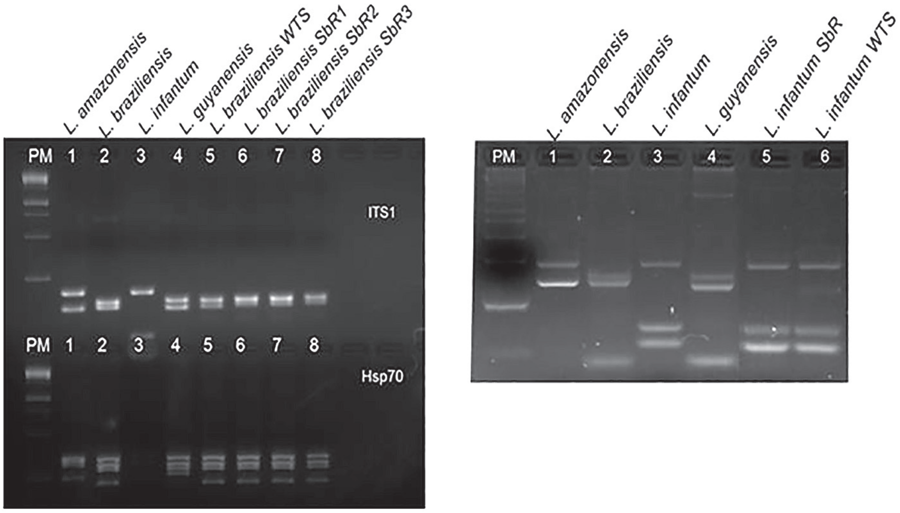

(A) Genotyping of wild-type (WT) and Sb^{III}-resistant *Leishmania braziliensis* using ITS1 and Hsp70 markers. (B) Genotyping of WT and Sb^{III}-resistant *L. infantum* using ITS1 marker. Polymerase chain reaction (PCR) products from ITS1 and HSP70 genes were digested with restriction endonuclease *Hae*III, and the fragments separated by 4% agarose gel electrophoresis, stained with GelRed. The standard reference *Leishmania* species were used: *L. amazonensis*, *L. braziliensis*, *L. infantum*, and *L. guyanensis*. WTS represents the WT line and SbR represents the Sb^{III}-resistant line.
